# Directional sensitivity of the $A_{1g}$ phonon in biaxially strained bismuth heterofilms studied by transient white light reflectivity

**DOI:** 10.1063/4.0001200

**Published:** 2026-06-11

**Authors:** Fabian Thiemann, Maja Penkl, Michael Horn-von Hoegen

**Affiliations:** Department of Physics, University of Duisburg-Essen, Lotharstrasse 1, 47057 Duisburg, Germany

## Abstract

Strain engineering in epitaxial Bi(111) films on Si(111) enables temperature-tunable phonon frequencies through thermal expansion mismatch. Using *in situ* low-energy electron diffraction combined with broadband femtosecond transient reflectivity on the same samples, we directly correlate anisotropic lattice parameters with the redshift of the coherent $A_{1g}$ phonon mode. The in-plane lattice parameter remains locked to the Si substrate and is therefore nearly temperature-independent, imposing strong biaxial compressive strain, causing an additional expansion of the Bi lattice along the *c*-axis. By applying an extended phonon-shift model, we disentangle the contributions of intrinsic thermal expansion, thermal-mismatch strain, and anharmonicity to the phonon softening. The enhanced redshift observed in strained films cannot be accounted for by expansion or mismatch alone; instead, it arises from a strengthened anharmonic term that additionally scales with strain along the *c*-axis, being insensitive to the in-plane lattice parameter and thereby enabling targeted control of phonon frequencies.

## I. INTRODUCTION

Manipulating the lattice structure of solids in general affects their electronic and phononic band structures and thereby the bandgap, effective mass, electron–phonon coupling and optical properties. These modifications arise from changes of key parameters, like bond lengths, symmetry, and anisotropic lattice expansion, that are governed by the underlying interatomic force constants. The resulting new vibrational properties manifest directly in changes of phonon frequencies measured by Raman spectroscopy or through ultrafast pump-probe techniques. Strain engineering offers a clean and reversible way to manipulate the lattice parameters and anisotropic distortion, and therefore its influence on phonon dispersion and frequency without changing the material's composition,^1–4^ thus directly targeting specific resonances, modifying electron–phonon interactions and controlling optical responses. Through such targeted control, strain-engineering strategies support a broad range of emerging applications, including ultrafast optical switching,^5^ thermoelectrics,^6,7^ optoelectronics,^8^ strain sensing,^9^ and nonlinear phononics.^10^

The semimetal bismuth (Bi) with its anisotropic A7 rhombohedral structure is particularly prone to structural manipulation due to its inherent Peierls distortion, low anisotropic effective masses, and strong electron–phonon coupling.^11–14^ Optical phonons along the [111]-direction (c-axis), parallel to the Peierls distortion, are especially sensitive to lattice changes.^15^ Among them, the $A_{1g}$ phonon mode at the Γ point can be easily excited by femtosecond laser pulses via the displacive excitation of coherent phonons (DECP) mechanism^16–27^ or detected by Raman spectroscopy.^28–30^

Strain engineering in bismuth can be readily achieved by growing Bi heterofilms on suitable substrates. The growth conditions of bismuth are highly tunable, allowing precise control over strain, stress, defect densities, and thin-film effects—key parameters for tailoring its phononic behavior. Monocrystalline epitaxial Bi films thus provide an excellent platform for strain engineering, where film thickness, substrate type and orientation, and growth temperature can be systematically adjusted to tune lattice distortions.^31–42^

Here, we use Bi(111) films grown on Si(111) substrates, which experience biaxial strain in the surface plane due to lattice mismatch and differences in thermal expansion coefficients of the two materials.^39^ This strain compresses the in-plane lattice parameter *a* and induces tensile strain along the out-of-plane c-axis by tetragonal distortion, strongly affecting the $A_{1g}$ phonon mode. Additionally, the large mismatch in thermal expansion between Bi and Si causes the strain to vary strongly with temperature, providing an additional tuning parameter. Notably, this substrate-induced strain persists not only in thin films but also in thick monocrystalline layers, extending over several tens of nanometers.

Previous studies of temperature-dependent phonons in bismuth have examined bulk crystals,^22,43^ polycrystalline films,^28^ or thin monocrystalline films.^30^ Bulk samples exhibit no substrate-induced strain, whereas thin films, as demonstrated by Hoff *et al.*,^27^ are additionally affected by carrier confinement effects that strongly affect the phonons' frequency. To directly assess the effect of biaxial in-plane strain without interference of confinement effects, we investigate thick Bi films *in situ* over a wide temperature range.

Phenomenological models describing the temperature dependence of phonon frequencies are well established in the literature^44–48^ and have been successfully applied to materials ranging from atomically thin two-dimensional layers^49,50^ to bulk crystals.^51^ In their general form, these models incorporate a contribution from thermal expansion^48^ and a term accounting for multi-phonon interactions.^47^ Depending on the material and the specific phonon mode, either term may dominate the overall temperature dependence. Recently, such models have been applied to thin Bi films in the work by Lu *et al.*^30^ However, their study did not include the mismatch-strain component nor any explicit measurement of the lattice parameters as a function of temperature, leaving open questions about the individual contributions in strained epitaxial Bi films.

Here, we use femtosecond optical pump-probe techniques to track the temperature dependence dΩ/dT of the $A_{1g}$ phonon frequency Ω(T), and relate it to anisotropic lattice parameter changes measured by electron diffraction. Both high-resolution spot profile analysis low-energy electron diffraction (SPA-LEED) and broadband femtosecond transient reflectivity (bb-fs-TR) measurements were performed *in situ* in an ultrahigh-vacuum chamber on the same samples. The phonon frequencies show the expected redshift with increasing temperature, with a steeper slope dΩ/dT than in relaxed samples^22,28^ and a lower overall frequency than in thin-film studies^30^—the latter agrees with confinement effects reported by Hoff *et al.*^27^ We analyze these results using the established phonon-shift models, which we extend to include a thermal expansion-mismatch term,^49–51^ and compare our fits to literature data.^22,28^ We find that the multi-phonon-interaction term additionally scales with strain along the *c*-axis. This identifies the structural parameters that govern phonon tuning in bismuth and leads to potential strategies for targeting them in future strain-engineering applications.

## II. EXPERIMENTAL DETAILS

All experiments were conducted under ultrahigh-vacuum (UHV) conditions (base pressure $<2\cdot 10^{-10}\text{ mbar}$) to ensure a stable and contamination-free environment for the Bi films. All Bi films were grown in a homebuilt molecular beam epitaxy (MBE) system equipped with a SPA-LEED for structural characterization. The Bi film thickness was monitored *in situ* during growth by recording layer-by-layer intensity oscillations of the (00) spot in diffraction, using an external electron gun. The internal gun of the SPA-LEED was used to obtain high-resolution diffraction patterns of the surface of the Si substrate and Bi films. Sample temperatures were controlled between 80 and 500 K using a continuous flow cryostat with an integrated resistance wire heater, connected to a feedback control loop (Lake Shore Cryotronics). Additional direct-current heating was used for applying rapid temperature changes. The temperature was measured using a silicon diode in the low-temperature regime and a pyrometer (Impac IGA 12) at higher temperatures. The UHV-system is equipped with fused-silica windows for *in situ* bb-fs-TR measurements. The bb-fs-TR setup is driven by an amplified Yb:YAG laser (Light Conversion Carbide CB5-SP) with a pulse energy of 100 μJ and a duration of 169 fs at a repetition rate of 5 kHz. We use the second harmonic (SH) of the laser output, centered around 515 nm for pumping the samples and supercontinuum white light pulses in the range of 500−950 nm to probe the pump-induced reflectivity change $\Delta R/R_{0}$ at a time delay Δt with a spectrometer (Avantes AvaSpec-3648).

**FIG. 1.**
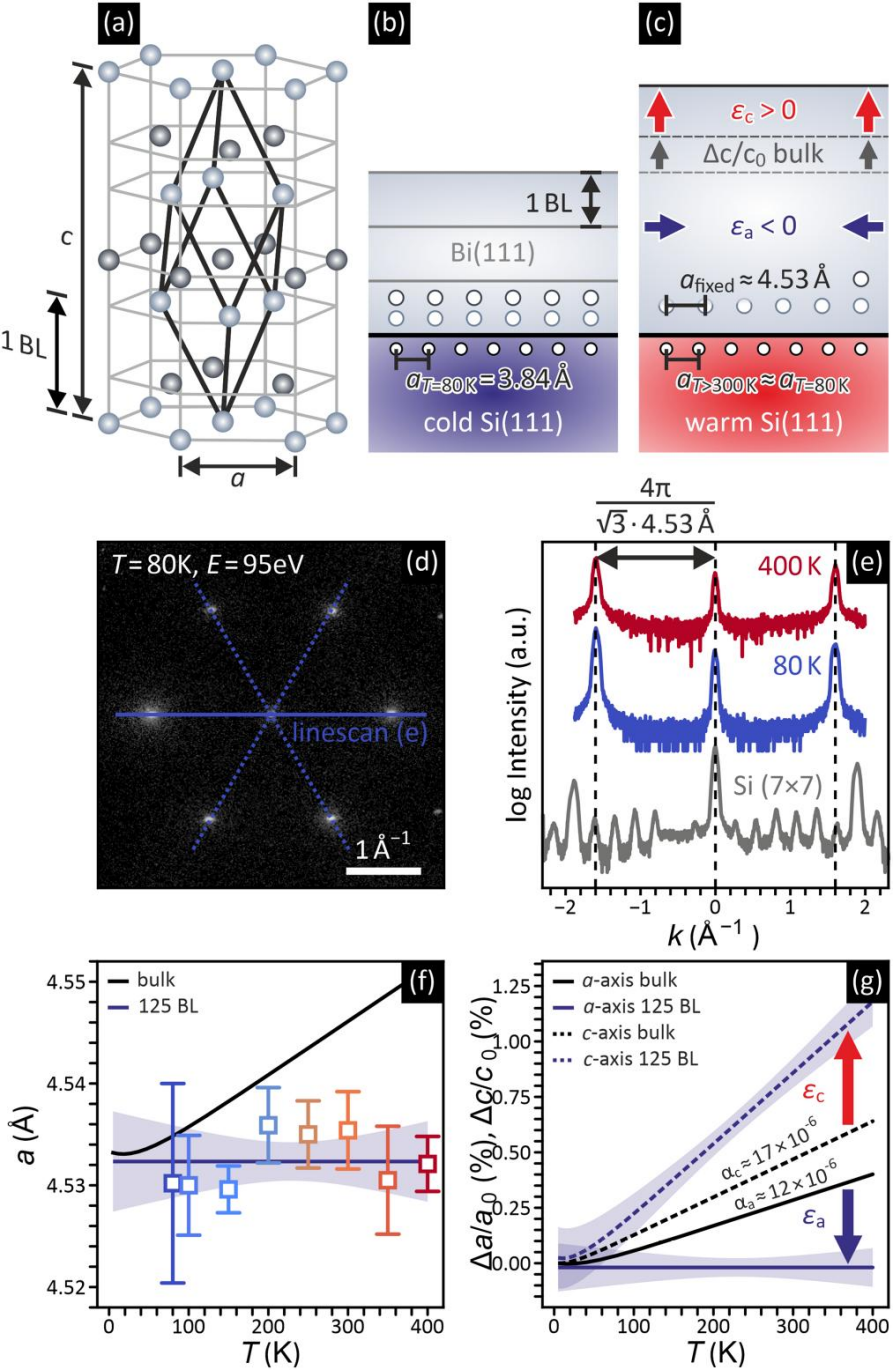
(a) Schematic representation of bismuth's unit cell with in-plane and out-of-plane lattice parameters *a* and *c*, respectively. (b) Sketch of a cold heterofilm sample with the characteristic lattice mismatch. (c) Warm heterofilm sample: The in-plane lattice parameter *a* remains constant while the *c*-axis expands due to tensile strain. (d) LEED-pattern of a 125 BL thick Bi film at 80 K. (e) Corresponding LEED intensity profiles for the film in (d) at 80 and 400 K compared to that of silicon's (7×7)-surface reconstruction. The positions of the first-order diffraction spots does not change over this temperature range. (f) Temperature dependence of lattice parameter *a* for the 125 BL film (blue) and expected value for bulk (black) modeled from thermal expansion parameters.^52^ (g) Relative lattice expansion of the *a*- and *c*-axes for 125 BL and bulk. The σ confidence bands are marked by blue areas in (f) and (g).

Silicon wafers with [111]-orientation (As-doped, 4- 6 mΩcm, ±0.1° tolerance) were used as substrates. Prior to film deposition, the substrates were cleaned with ethanol, degassed at 600 °C, and subject to repeated flash-annealing cycles at 1250 °C to remove the native oxide layer and other impurities. After cleaning, LEED patterns showed the characteristic ( 7×7)-reconstruction of the bare Si(111) surface. Bismuth grows epitaxially in the [111]-direction on Si(111) substrates.^31,33^ Because of its A7-rhombohedral structure, it exhibits alternating interlayer distances [Fig. 1(a)]. The film thickness is therefore expressed in bi-layers (BLs), where 1 BL=3.94  Å. Bismuth (6N from MaTeck) was evaporated from a thermal effusion cell onto the bare Si(111) surface at a rate of 0.5 BL/min while keeping the substrate at 150 K. The film thickness was monitored *in situ* via BL-intensity oscillations of the SPA-LEED (00)-spot.^38^ Bi films with thickness from 6 to 125 BL have been grown. Subsequently, the Bi films were annealed at 400 K, and their crystalline quality was examined using SPA-LEED, which provides high reciprocal space resolution [Fig. 1(c)]. The sharp and intense first-order diffraction spots confirm that Bi films are well-oriented and monocrystalline.

## III. RESULTS AND DISCUSSION

### A. Lattice parameters and induced strain

Figure 1 summarizes the LEED results for determination of the in-plane lattice parameter *a*. Panel (d) shows a representative SPA-LEED pattern for a 125 BL thick Bi film at 80 K. Panel (e) presents the corresponding intensity line profiles through the first-order integer spots along the direction indicated in panel (d) at a temperature of 80 and 400 K, together with a profile from a bare Si(111) surface, exhibiting its characteristic (7×7)-surface reconstruction. For each temperature, the distances *b* from the (00)-spot to the first-order diffraction spots were averaged over all three sets of spots ( 0°, 60°, and 120°). The in-plane lattice parameter was then obtained via $a=4\pi /(b\sqrt{3})=4.532\;Å$ and plotted as open squares in Fig. 1(f). Within the error margins, there is no temperature dependence of the in-plane lattice parameter *a*, which is consistent with earlier results for 5 and 50 BL films above 300 K.^39^ According to the work by Payer *et al.*, the value of *a* depends on the film thickness and can be tuned by adjusting the annealing temperature $T_{a}$.^39^ Here, we chose $T_{a}=400\;K$ such that *a* matches the bulk value of Bi at T→0, i.e., $a_{0}=4.532\;Å$. To compare the thermal evolution of $a_{125\text{BL}}(T)$ of the 125 BL film to those of Bi bulk, we used literature data recorded at 298 K^53^ together with a first-principles thermal expansion model by Arnaud *et al.*^52^ to reconstruct the expected bulk behavior. The bulk $a_{\text{bulk}}(T)$ is plotted in black in Fig. 1(f). The 125 BL heterofilm exhibits a clear deviation from the bulk behavior, with compressive in-plane strain $\varepsilon_{a}(T)=\left(a_{125\text{BL}}(T)-a_{\text{bulk}}(T)\right)/a_{\text{bulk}}(T)$ increasing almost linearly with temperature.

The temperature-independent in-plane lattice parameter *a* in epitaxial Bi/Si(111) films^39^ results from the bismuth lattice locking into registry with the Si substrate with its almost negligible thermal expansion of +0.06% between 80 and 400 K.^54^ This registry results in a commensurate match of 11 Bi to 13 Si atoms. Because Bi itself exhibits a large thermal expansion coefficient above 80 K,^52,55–57^ it would naturally expand upon heating; its inability to do so on the Si substrate therefore unavoidably induces biaxial compressive strain $\varepsilon_{a}$ in the (111)-plane of the film. As the Bi lattice is relaxed at T= 80 K, the in-plane compressive strain increases with temperature as illustrated by the solid lines in Fig. 1(g) through comparison of the relative lattice expansion $\Delta a/a_{0}\left(T\right)$ of the strained Bi film with bulk bismuth. This expansion is 0.40% at 400 K and thus an order of magnitude larger than the substrate's expansion. The difference between film and bulk directly corresponds to the compressive in-plane strain $\varepsilon_{a}\left(T\right)$ indicated by the blue arrow.

The elastic response of the Bi film to the biaxial strain $\varepsilon_{a}$ results in tensile strain $\varepsilon_{c}$ along the [111]-direction, i.e., along the *c*-axis. As a consequence, c(T) exhibits an enhanced expansion, exceeding the intrinsic anisotropic thermal expansion [Fig. 1(g)], and thereby exhibiting a significant increase of the interlayer spacing relative to bulk Bi. This behavior is consistent with prior observations of anisotropic strain effects in Bi films of varying thickness, where the interplay between *a* and *c* parameters was reported.^27^ The effect is illustrated schematically in Figs. 1(b) and 1(c) with a constant in-plane spacing *a*—constrained by the 11:13 match between the film and substrate lattices—and the enhanced out-of-plane expansion under tensile strain at higher temperatures. Direct measurement of the *c*-axis lattice parameter is not possible in our case, as LEED is mostly sensitive to the structures parallel to the surface plane. To overcome this limitation, we estimate c(T) indirectly by calculating the strain ratio $\nu_{\text{eff}}=-\varepsilon_{c}/\varepsilon_{a}$ in the case of biaxial strain $\varepsilon_{1}=\varepsilon_{2}=\varepsilon_{a}$ derived from Hooke's law in Voigt notation,^58,59^

$$\varepsilon_{i}=S_{ij}\sigma_{j}.$$ (1)

Because $\nu_{\text{eff}}$ links strain in different directions, it is called the biaxial or effective Poisson's ratio, and it may take negative values or values larger than 0.5.^60,61^ Since the elastic compliance tensor $S_{ij}$ of Bi^62–64^ and in-plane strain $\varepsilon_{a}$ are known, we can cancel out the stress components $\sigma_{j}$ in Eq. (1) and solve for

$$\nu_{\text{eff}}=-\frac{\varepsilon_{c}}{\varepsilon_{a}}=-\frac{\varepsilon_{3}}{\varepsilon_{1}}=-\frac{2S_{13}}{\left(S_{11}+S_{12}\right)}=1.28.$$ (2)

This allows c(T) to be calculated for the measured a(T) using

$$c=c_{\text{bulk}}\left[1-\nu_{\text{eff}}\;\frac{a-a_{\text{bulk}}}{a_{\text{bulk}}}\right],$$ (3)

where $a_{\text{bulk}}$ and $c_{\text{bulk}}$ are the lattice parameters of unstrained bulk at a given *T*. The calculated relative expansion $\Delta c/c_{0}(T)$ for our films is shown in Fig. 1(g) as dashed lines, enabling direct comparison to the expected bulk thermal expansion. The blue areas highlight the σ confidence bands for the relative expansion obtained from an uncertainty propagation of the uncertainties $\sigma_{a}(T)$ of the $a_{125\text{BL}}(T)$ data in Fig. 1(f). The uncertainty for the strain $\sigma_{\varepsilon ,c}$ is equivalent to the uncertainty $\sigma_{c,\text{rel}}$ of $\Delta c/c_{0}(T)$. Analogous to $\Delta a/a_{0}$, we obtain the strain $\varepsilon_{c}(T)$ by comparing the film to the bulk curves in panel (g). The observed strain values at 400 K of $\varepsilon_{c}\approx 0.54\;\%$ and $\varepsilon_{a}\approx -0.40\;\%$ are comparable to other studies on biaxial strain.^65,66^ At 400 K, the intrinsic thermal expansion of unstrained bulk Bi accounts for $\Delta c_{\text{bulk}}/c_{0}\approx 0.64\%$. In the anisotropically strained films, the compressive in-plane strain causes tetragonal distortion, leading to a further *c*-axis expansion of $\varepsilon_{c}\approx 0.54\;\%$, comparable in magnitude to the intrinsic thermal expansion. This extra *c*-axis lattice change is therefore expected to significantly affect the $A_{1g}$ phonon mode, whose atomic displacement occurs along this crystallographic direction. In Sec. III B, we examine this relation by measuring the coherent $A_{1g}$ phonon's frequency using femtosecond pump-probe transient reflectivity and linking the observed shifts to the strain‐induced lattice distortion.

### B. Measurement of optical phonons

**FIG. 2.**
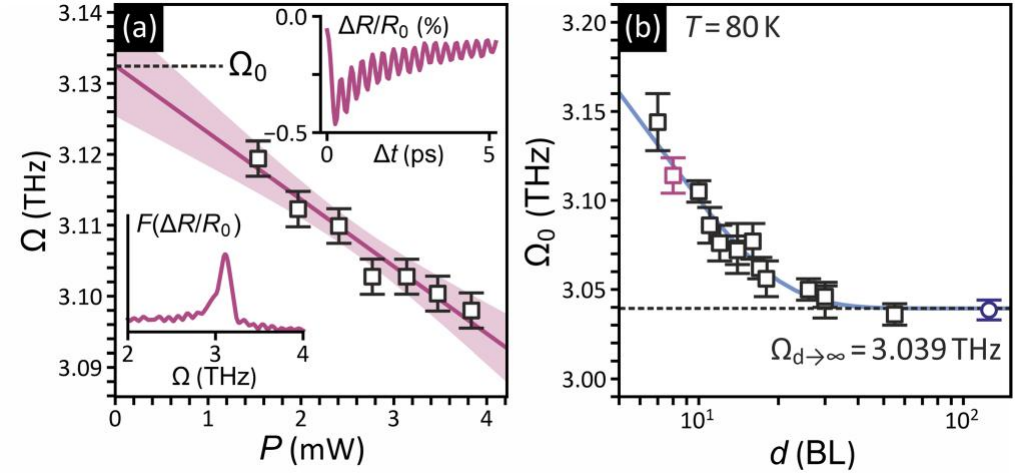
(a) Redshift of the $A_{1g}$ phonon frequency Ω for an 8 BL film for increasing pump-laser powers *P*. The insets show the observed oscillatory transient reflectivity $\Delta R/R_{0}$ vs Δt for a spectral range of λ=600 nm ± 15 nm at P=3.8 mW. The Fourier transform $F(\Delta R/R_{0})$ yields the $A_{1g}$ phonon frequency Ω. Extrapolation P→0 gives $\Omega_{0}$. (b) Frequencies $\Omega_{0}\left(d\right)$ as a function of Bi film thickness *d* exhibit a significant blueshift due to the confinement effect for d→0.^27^

**FIG. 3.**
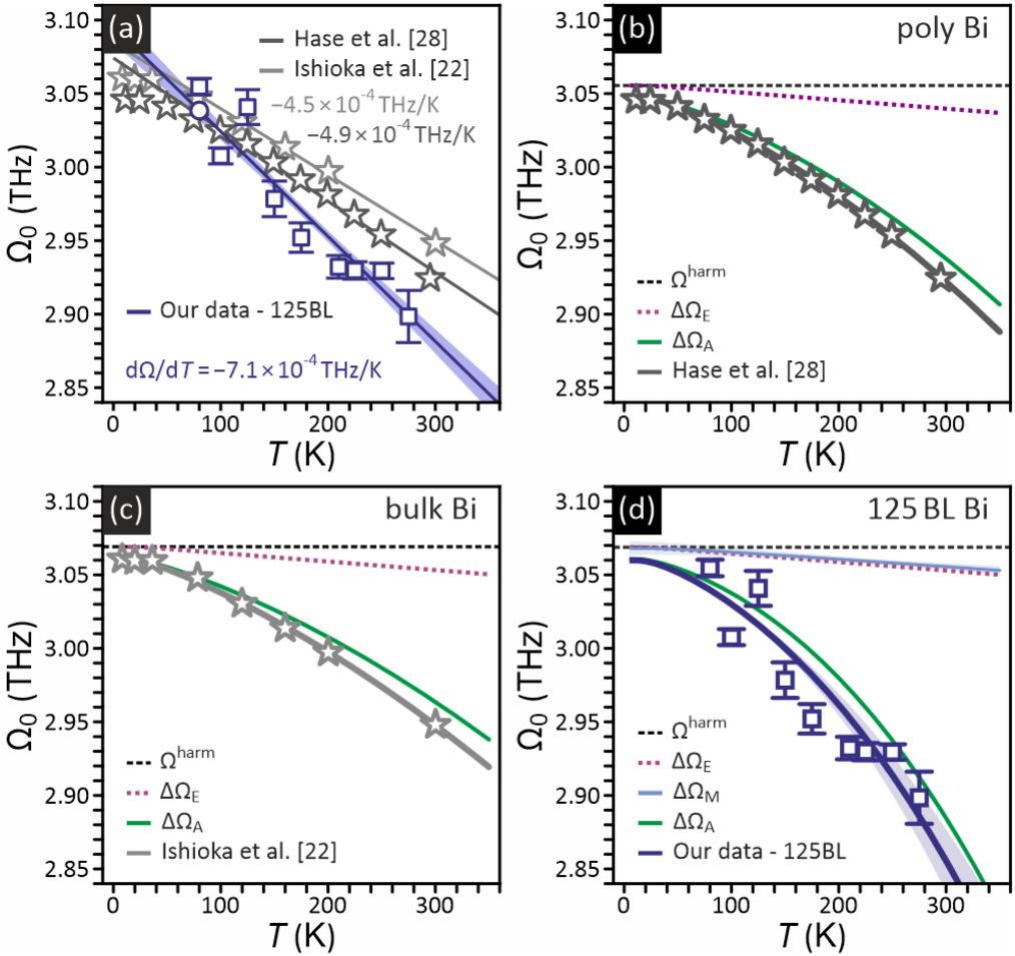
(a) Temperature dependence of frequency $\Omega_{0}(T)$ for the 125 BL film (blue squares) in comparison to data from literature for thick polycrystalline films (dark stars) [Adapted with permission from Hase *et al.*, Phys. Rev. B **58**, 5448–5452 (1998). Copyright 1998 American Physical Society.]^28^ and a bulk crystal (light stars) [Adapted from Ishioka *et al.*, J. Appl. Phys. **100**, 093501 (2006) with permission from AIP Publishing].^22^ (b) and (c) Application of the phonon softening model (4) to polycrystalline film and bulk data. The T→0 frequency $\Omega^{\text{harm}}$ is plotted as a reference as dashed black line. The components $\Delta \Omega_{E}\left(T\right)$ (magenta dashed line) and $\Delta \Omega_{A}\left(T\right)$ (green solid line) differ significantly in their contribution. (d) Application of the model to the 125 BL film. Due to the anisotropic mismatch strain, $\Delta \Omega_{M}\left(T\right)$ (light blue line) is added.

Figure 2 summarizes our bb-fs-TR measurements and the extracted $A_{1g}$ phonon frequencies. All measurements were performed using super-continuum white-light probe pulses, which prevent wavelength-dependent sign flips in the transient reflectivity signal that would arise by thin-film interference effects.^67^ As an example, the top-right inset of Fig. 2(a) shows the characteristic oscillatory modulation of $\Delta R/R_{0}$ as a function of pump-probe delay Δt for an 8 BL Bi film. The probe signal was spectrally averaged over λ=600 nm ± 15 nm, while the sample was excited at a pump power P=3.8 mW. The pulses were focused on a 840 μm spot with a repetition rate of 5 kHz. This results in a fluence range of 0.08– $0.13\;{\text{mJ}/\text{cm}}^{2}$ used in this work. Other studies have extracted the phonon frequency by fitting a damped oscillator model^17,19,21,25,43,68,69^ with a chirp $\Omega^{\prime }=\Omega +\beta t$. Because our fluences are comparatively small, we omitted the analysis of the chirp and determined the frequency Ω directly from the signal via Fourier transformation,^70,71^ as shown exemplarily in inset on the bottom left with a clear peak at Ω=3.098 THz. For bismuth, it is well known that the interatomic potential—and consequently the phonon frequency—softens linearly with increasing photo-excitation density.^15,19,23,68^ To enable direct comparison with Raman spectroscopy data from the literature, we measured Ω for several pump powers *P* and extrapolated Ω(P) linearly to P→0, shown in Fig. 3(a). This extrapolation eliminates different slopes of the phonon redshift caused by homogenization of excited carrier excitation densities up to 60 nm, beyond the optical penetration depth of $d_{\text{opt}}\approx 15\text{ nm}$.^69,71,72^ The resulting extrapolated values, denoted $\Omega_{0}$, represent the unperturbed $A_{1g}$ phonon frequencies and are shown in Fig. 3(b) for different film thicknesses ranging from 8 to 125 BL at T=80 K.

The measured thickness dependence of $\Omega_{0}(d)$ exhibits an exponential blueshift for films thinner than ∼50 BL. For thicker films, $\Omega_{0}(d)$ converges to the asymptotic value $\Omega_{\infty }=3.039\text{ THz}$. This significant blueshift was recently reported for epitaxial Bi films by Hoff *et al.*,^27^ who attributed it to bond-hardening effects arising from the confinement of electronic states in thin layers. In the present work, our goal is to isolate phonon frequency shifts ΔΩ of the A_1g_ mode that arise exclusively from anisotropic strain induced by the thermal expansion of the substrate–film system and to compare these results with unstrained Bi samples reported in the literature. To eliminate confinement-related contributions, we therefore select a film thickness of 125 BL, where $\Omega_{0}(d)$ has already reached its bulk-like asymptotic value.^27^ For the 125 BL film, we repeated the $\Omega_{0}$ measurements of Fig. 2(a) over a temperature range from 80 to 280 K. Figure 3(a) shows the resulting $\Omega_{0}(T)$ data (open squares), together with data for polycrystalline films from Hase *et al.*^28^ (dark stars) and bulk crystals from Ishioka *et al.*^22^ (light stars). The monocrystalline strained films exhibit a noticeably larger slope $d\Omega_{0}/dT$ compared to the unstrained bulk or polycrystalline references. This difference is highlighted by fitting a line to each of the three datasets as a guide to the eye. This observation raises the question of which structural parameters govern the thermal evolution of $\Omega_{0}\left(T\right)$. We use established phonon-shift models to quantify the contributions by thermal expansion, multi-phonon interactions, and mismatch-induced strain, revealing the key parameters that control $\Omega_{0}$.

### C. Phonon-shift model

Widely accepted models, originally developed to describe temperature-dependent Raman shifts, capture the thermal behavior of phonon frequencies.^44–48,73,74^ In their general form, these models decompose the total frequency shift into two contributions. The first is the intrinsic thermal expansion term ( $\Delta \Omega_{E}$) accounting for changes in the lattice parameters and their effect on the interatomic force constants. The second term ( $\Delta \Omega_{A}$) describes modifications of the phonon self-energy arising from multi-phonon interactions and lattice anharmonicity. More recent studies on two-dimensional layered materials^49,50^ and thick semiconductor films^51^ have extended these models by introducing an additional term ( $\Delta \Omega_{M}$) that considers strain effects originating from the mismatch in thermal expansion coefficients (TEC) of the film and its substrate. It describes how this anisotropic strain modifies the phonon frequency beyond its intrinsic thermal behavior. Earlier measurements by Hase *et al.*,^28^ Ishioka *et al.*,^22^ and Boschetto *et al.*^43^ reported the temperature evolution of the $A_{1g}$ phonon frequency in bulk and polycrystalline Bi films, but focused more on the dephasing of the coherent phonons rather than the modeling of its frequency. The work by Boschetto was performed at much larger incident fluences of $6\;{\text{mJ}/\text{cm}}^{2}$ and is therefore not comparable to our data because it exhibits strong phonon softening. Lu *et al.*^30^ applied the established phonon-shift model to ∼30 BL Bi films, describing their experimental data well without explicit treatment of strain from TEC mismatch. They found that the anharmonic contribution, particularly the three-phonon term, dominates the temperature dependence of $\Omega_{0}\left(T\right)$ in Bi. Interestingly, they also observed a larger thermal redshift, similar to that observed in our strained monocrystalline films. The absolute phonon frequencies in their thin-film samples were higher than in our work, most likely due to confinement effects absent in films thicker than d>50 BL. By extending their formalism to include the TEC mismatch term and a strain correction to $\Delta \Omega_{A}$, we develop a more general description that accounts for additional strain effects in epitaxial Bi films, enabling direct comparison between strained and unstrained systems. Accordingly, the phonon-shift model used in our work can be written in its general form as

$$\Omega_{0}\left(T\right)=\Omega^{\text{harm}}+\Delta \Omega_{E}\left(T\right)+\Delta \Omega_{M}\left(T\right)+\Delta \Omega_{A}\left(T\right).$$ (4)

Here, $\Omega^{\text{harm}}$ denotes the harmonic contribution to the phonon frequency at zero temperature. Since the T<80 K regime is inaccessible in our setup, $\Omega^{\text{harm}}$ must be inferred rather than measured. The SPA-LEED analysis reveals that the 125 BL film is almost strain‐free at T<80 K and thus exhibits the same lattice parameter as bulk Bi. Therefore, $\Omega^{\text{harm}}$ was estimated from $\Omega_{0}\left(T\to 0\right)-\Delta \Omega_{A}\left(T\to 0\right)$ reported for bulk Bi crystals by Ishioka *et al.*^22^ This was done in an iterative process since all terms in Eq. (4) depend on $\Omega^{\text{harm}}$. The model applied to the 125 BL film is shown in Fig. 3(d), and for comparison, the same model is applied to the literature data of Hase *et al.*^28^ and Ishioka *et al.*^22^ in Figs. 3(b) and 3(c). The thermal expansion component of the model^48^ is given by

$$\Delta \Omega_{E}(T)=\Omega^{\text{harm}}\text{ exp}\left(-\gamma \int_{0}^{T}\alpha_{c}(T^{\prime })\;dT^{\prime }\right)-\Omega^{\text{harm}},$$ (5)

with γ the anisotropic Grüneisen parameter of Bi and $\alpha_{c}$ the anisotropic thermal expansion coefficient along the *c*-axis, both obtained from first‐principles calculations.^52^ The resulting $\Delta \Omega_{E}\left(T\right)$ curves are plotted in magenta in Figs. 3(b)–3(d). Consistent with the findings of Lu *et al.*,^30^ $\Delta \Omega_{E}\left(T\right)$ is small, contributing only ≈10 % to the total phonon redshift. The TEC mismatch or strain contribution is described by

$$\Delta \Omega_{M}(T)=-\Omega^{\text{harm}}\gamma \;\varepsilon_{c}(T),$$ (6)

with the same Grüneisen parameter γ as in Eq. (5), but linked to the additional strain $\varepsilon_{c}$ along the c-axis—induced by the commensurate 11:13 registry of the film to the substrate—instead of the intrinsic thermal expansion. This strain $\varepsilon_{c}\left(T\right)$ is extracted from Fig. 1(g) as the difference in relative *c*-axis expansion between the strained film and bulk Bi. The resulting $\Delta \Omega_{M}\left(T\right)$ curve is plotted in light blue in Fig. 3(d). Its magnitude is comparable to that of $\Delta \Omega_{E}$, yet still far too small to account for the ≈40 % larger redshift observed in strained films compared to bulk references. The anharmonic contribution is modeled using the phenomenological expressions of Klemens, Hart *et al.*, and Balkanski *et al.*,^46,47,74^

$$\begin{array}{cc} \Delta \Omega_{A}(T)=A\left[1+\frac{2}{e^{x}-1}\right]+B\left[1+\frac{3}{e^{y}-1}+\frac{3}{{\left(e^{y}-1\right)}^{2}}\right], & \end{array}$$ (7)

where *A* and *B* are fit parameters, and $x=\hbar \Omega^{\text{harm}}/2k_{B}T$ and $y=\hbar \Omega^{\text{harm}}/3k_{B}T$ the arguments used in the Bose factors describing the occupation for three- and four-phonon interactions. The challenge with this model arises from the temperature-dependent strain $\varepsilon_{c}(T)$ observed in our data. In a simplified framework, the parameters *A* and *B* represent the frequency shift at zero temperature, driven by changes in the zero-point energy. At T→0, where strain is absent, $\Delta \Omega_{A}(0\;K)=A+B$ should be identical for both the unstrained literature data and our films. However, because the Bose factors remain unchanged, the larger redshift observed in strained films compared to the model persists, as shown by $\Delta \Omega_{A,125\text{BL}}(T)\neq \Delta \Omega_{A,\text{bulk}}(T)$ in Fig. 3.

Because *A* and *B* depend on the phonon's self-energy and thereby on the interatomic potential,^73^ which is modified by strain, we have to introduce a strain correction $\Delta \Omega_{A}^{\prime }=C(\Delta \Omega_{A},\varepsilon_{c})$. Deriving a general description for this correction is complicated and not necessary for modeling our data; therefore, we approximate it using the approximation for the *A* and *B* parameters used by Klemens,^74^ who scales these parameters by a factor ${\left(\alpha -\beta \right)}^{2}/{\left(\alpha +\beta \right)}^{2}$, containing the force constants α and β of the harmonic potential. With present tensile strain, they are reduced by $\alpha^{\prime }=\alpha -d\cdot \varepsilon_{c}$ and $\beta^{\prime }=\beta -e\cdot \varepsilon_{c}$. The linear term results in a correction factor $\Delta \Omega_{A}^{\prime }=\Delta \Omega_{A}\left(1+C\cdot \varepsilon_{c}\right)$ with a positive *C*. The model changes to

$$\begin{array}{cc} \Delta \Omega_{A}(T)=(1+C\cdot \varepsilon_{c})(A\left[1+\frac{2}{e^{x}-1}\right] & \\ +B\left[1+\frac{3}{e^{y}-1}+\frac{3}{{\left(e^{y}-1\right)}^{2}}\right]). & \end{array}$$ (8)

**FIG. 4.**
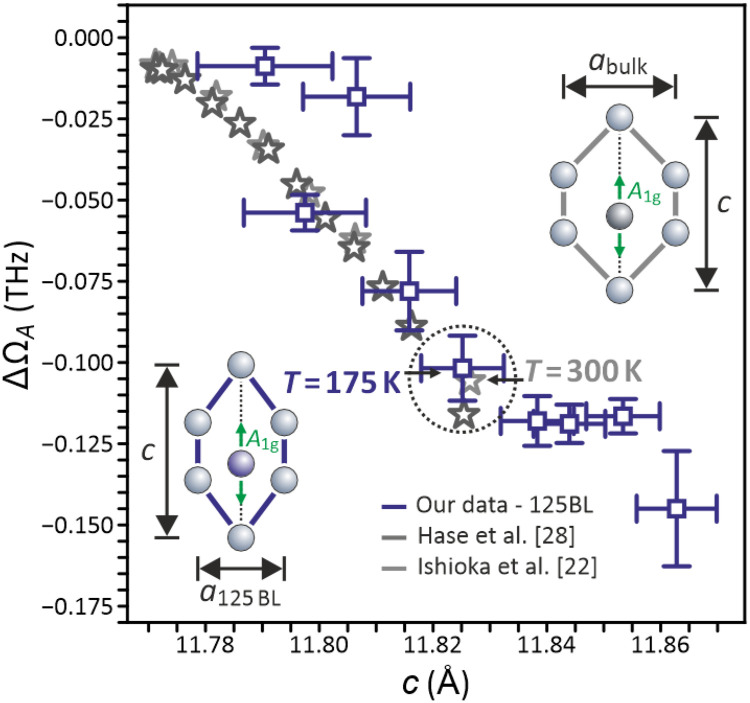
Dependence of the anharmonic frequency shift component $\Delta \Omega_{A}$ on the out-of-plane lattice parameter *c* in comparison to data from literature [Adapted with permission from Hase *et al.*, Phys. Rev. B **58**, 5448–5452 (1998). Copyright 1998 American Physical Society;^28^ and from Ishioka *et al.*, J. Appl. Phys. **100**, 093501 (2006) with permission from AIP Publishing^22^]. The frequency shifts collapse on a single curve although the in-plane lattice parameter a(c) and temperature T(c) are different for the datasets. This is highlighted for the datapoints at c≈11.823  Å encircled by the dotted ellipse together with sketches of their unit cells' projection (not to scale) onto the a–c plane.

To apply this model, we first fitted the literature data from Hase *et al.*^28^ and Ishioka *et al.*,^22^ extracting the parameters *A* and *B*. Using the average of these values, we then fitted our experimental data using only the parameter *C*. The uncertainties in *A* and *B* result from the previous fits, whereas the uncertainty of *C* was approximated using a Monte Carlo method,^75^ with $\sigma_{A}$, $\sigma_{B}$, and $\sigma_{\varepsilon ,c}\left(T\right)$ as input parameters. As illustrated by the green and blue curves in Fig. 3(d) and Table I, the model accurately captures our data across the measured temperature range. However, at higher temperatures approaching the melting point, the $\left(1+C\cdot \varepsilon_{c}\right)$ approximation may break down due to significant changes in the interatomic potential. This analysis demonstrates that the standard model in Eq. (7) is insufficient to describe phonon shifts in strained samples and that adding a correction term is essential. Plotting the extracted frequency shifts $\Delta \Omega_{A}(T)$ as a function of the *c*-axis lattice parameter c(T)—rather than temperature *T*—collapses all datasets onto a single curve, as shown in Fig. 4. This collapse is likely coincidental and applies specifically to the temperature range investigated, as shown in Eq. (8). Here, $\Delta \Omega_{A}(T)$ depends nonlinearly on *T* but is linear in c(T) and $\varepsilon_{c}(T)$. Despite their distinct origins—thermal occupation and strain—the two shift mechanisms in $\Delta \Omega_{A}$ exhibit similar scaling with the *c*-axis lattice parameter. Consequently, strain-induced shifts can reach magnitudes comparable to those arising from occupation effects. This is consistent with the $A_{1g}$ phonon's atomic displacement along the [111]-direction, which renders this mode highly responsive to variations in both interlayer and intralayer spacing arising from the Peierls distortion. Variations of the in-plane lattice parameter *a* have no measurable effect on the frequency of the $A_{1g}$ phonon mode, as shown in Fig. 4 by the data points encircled by the dotted ellipse at c≈11.823 Å. The 125 BL film and bulk Bi exhibit the same frequency shift $\Delta \Omega_{A}$, despite having different in-plane lattice parameters and temperatures, and the shifts are caused by different mechanisms: $a_{125\text{BL}}(175\;K)=4.532\;Å$ compared with $a_{\text{bulk}}(300\;K)=4.546\;Å$.This insensitivity to the in-plane lattice parameter suggests that the $A_{1g}$ mode couples only weakly—if at all—to lateral strain. Thus, selective tuning of c(T) enables precise strain engineering of the $A_{1g}$ phonon frequency—and potentially other phononic properties—in the same order of magnitude as occupation driven multi-phonon interactions.

**TABLE I.** Overview of fit results for different datasets.

	*A* ( $10^{-3}\text{ THz}$)	*B* ( $10^{-4}\text{ THz}$)	*C*
Our data	−7.33 ± 0.42	−4.5 ± 0.3	167 ± 50
Ishioka *et al.*^22^	−7.72 ± 0.73	−3.7 ± 0.5	
Hase *et al.*^28^	−6.94 ± 0.44	−5.3 ± 0.3	

## IV. SUMMARY AND CONCLUSIONS

In summary, epitaxial Bi(111) films were grown on Si(111) substrates under ultrahigh-vacuum conditions and characterized *in situ* using low-energy electron diffraction and broadband femtosecond transient reflectivity. This combined approach enabled a direct correlation between structural parameters and the temperature evolution of the $A_{1g}$ phonon mode. Diffraction reveals that the in-plane lattice parameter ( a=4.532 Å) of the Bi films is temperature-independent because the Bi lattice is locked in commensurate registry to the Si(111) substrate, preventing its intrinsic thermal expansion and thereby imposing a temperature-dependent biaxial strain. Transient reflectivity shows that the $A_{1g}$ phonon frequency in strained Bi films exhibits a significantly larger thermal redshift than in unstrained reference samples. We applied a phonon-shift model to the measured temperature dependence, enabling us to decompose the total frequency shift into three distinct contributions originating from thermal lattice expansion, lattice mismatch-induced strain, and anharmonic multi-phonon interaction. In Bi, the anharmonic term is the dominant contribution to the observed phonon frequency redshift. The comparison between strained films and literature data for unstrained samples reveals that this anharmonic term consist of a phonon-occupation and a strain component, which scales the same with the *c*-axis lattice parameter. This large anharmonic-strain component highlights the remarkable directional selectivity of the $A_{1g}$ phonon, which is highly sensitive to the intra- and interlayer spacing along [111], yet essentially insensitive to lattice changes within the (111) plane.

## Data Availability

The data that support the findings of this study are available from the corresponding author upon reasonable request.
